# Anti-Classical Swine Fever Virus Strategies

**DOI:** 10.3390/microorganisms9040761

**Published:** 2021-04-06

**Authors:** Jindai Fan, Yingxin Liao, Mengru Zhang, Chenchen Liu, Zhaoyao Li, Yuwan Li, Xiaowen Li, Keke Wu, Lin Yi, Hongxing Ding, Mingqiu Zhao, Shuangqi Fan, Jinding Chen

**Affiliations:** 1 College of Veterinary Medicine, South China Agricultural University, Guangzhou 510642, China; fanjindai@stu.scau.edu.cn (J.F.); yxliao@soil.gd.cn (Y.L.); zmr15625156296@stu.scau.edu.cn (M.Z.); liuchenchen@stu.scau.edu.cn (C.L.); lizhaoyao@stu.scau.edu.cn (Z.L.); waner20191028012@stu.scau.edu.cn (Y.L.); xiaowenlee@stu.scau.edu.cn (X.L.); wukeke@stu.scau.edu.cn (K.W.); yilin@scau.edu.cn (L.Y.); dinghx@scau.edu.cn (H.D.); zmingqiu@scau.edu.cn (M.Z.); 2 Guangdong Laboratory for Lingnan Modern Agriculture, College of Veterinary Medicine, South China Agricultural University, Guangzhou 510642, China; 3 Key Laboratory of Zoonosis Prevention and Control of Guangdong Province, Guangzhou 510642, China

**Keywords:** classical swine fever virus, host protein, antiviral target, antiviral drug, RNA interference

## Abstract

Classical swine fever (CSF), caused by CSF virus (CSFV), is a highly contagious swine disease with high morbidity and mortality, which has caused significant economic losses to the pig industry worldwide. Biosecurity measures and vaccination are the main methods for prevention and control of CSF since no specific drug is available for the effective treatment of CSF. Although a series of biosecurity and vaccination strategies have been developed to curb the outbreak events, it is still difficult to eliminate CSF in CSF-endemic and re-emerging areas. Thus, in addition to implementing enhanced biosecurity measures and exploring more effective CSF vaccines, other strategies are also needed for effectively controlling CSF. Currently, more and more research about anti-CSFV strategies was carried out by scientists, because of the great prospects and value of anti-CSFV strategies in the prevention and control of CSF. Additionally, studies on anti-CSFV strategies could be used as a reference for other viruses in the Flaviviridae family, such as hepatitis C virus, dengue virus, and Zika virus. In this review, we aim to summarize the research on anti-CSFV strategies. In detail, host proteins affecting CSFV replication, drug candidates with anti-CSFV effects, and RNA interference (RNAi) targeting CSFV viral genes were mentioned and the possible mechanisms related to anti-CSFV effects were also summarized.

## 1. Introduction

**Figure 1:**
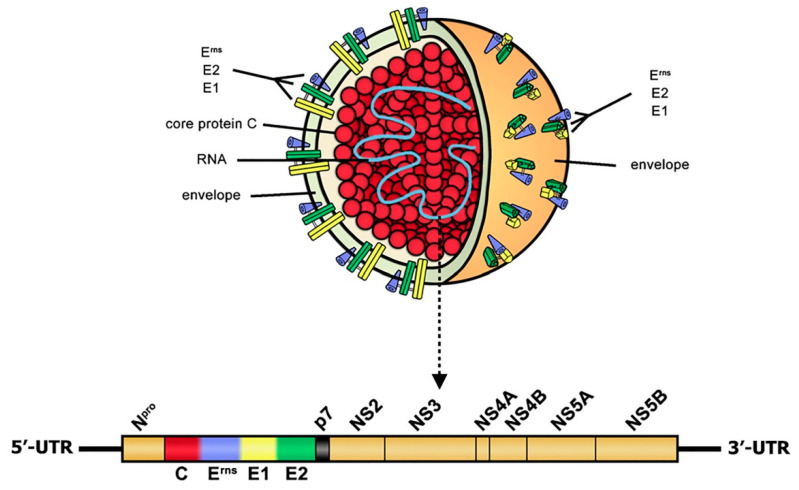
Schematic description of virion structure and genome organization of classical swine fever virus (CSFV) [4]. This figure comes from the literature reported by Beer et al. [4] with some modifications. CSFV is an enveloped, single-stranded, positive-sense RNA virus. Glycoproteins (E^rns^, E1, and E2) are located on the external part of viral particles and are important for viral infection. CSFV RNA genome consists of a single large open reading frame (ORF) flanked by a 5′-untranslated region (5′-UTR) and a 3′-UTR. A polypeptide precursor could be encoded by ORF of CSFV and then cleaved into four structural proteins (C, E^rns^, E1, and E2) and eight non-structural proteins (N^pro^, p7, NS2, NS3, NS4A, NS4B, NS5A, and NS5B).

Classical swine fever (CSF) is a highly contagious swine disease characterized by high fever, multiple hemorrhages, and gastrointestinal symptoms with high morbidity and mortality, which has caused significant economic losses to the pig industry worldwide [1]. CSF in wild boars is also endemic in some countries, which poses a serious threat to domestic pigs [2]. The causative agent of CSF is the CSF virus (CSFV), an important member of the *Pestivirus* genus within the Flaviviridae family. CSFV is an enveloped, single-stranded, positive-sense RNA virus containing a 12.3 kb RNA genome, which consists of a 5′-untranslated region (5′-UTR), a single large open reading frame (ORF), and a 3′-UTR. A polypeptide precursor could be encoded by ORF of CSFV and then cleaved into four structural proteins (C, E^rns^, E1, and E2) and eight non-structural proteins (N^pro^, p7, NS2, NS3, NS4A, NS4B, NS5A, and NS5B) through the processing of the polypeptide precursor by viral and cellular proteases (Figure 1) [1,3,4].

Based on partial sequences of CSFV 5′-UTR, E2, and NS5B, phylogenic typing of CSFV isolates worldwide was carried out. In general, CSFV strains could be classified into three genotypes (1, 2, and 3) and eleven subgenotypes (1.1–1.4, 2.1–2.3, and 3.1–3.4) [5,6]; subgenotype 2.1 isolates were further divided into three sub-subgenotypes (2.1a–2.1c) [7,8,9]. Because of the high genetic diversity of subgenotype 2.1 strains, Gong et al. [10] suggested dividing the subgenotype 2.1 strains into ten sub-subgenotypes (2.1a–2.1j). As reported, CSFV strains of genotype 1, 2, and 3 are all epidemic in the world, while strains of genotype 2 have gradually become dominant globally [11,12,13,14].

CSFV strains’ virulence, ranging from high, moderate, to low virulence, is the crucial determinant of CSFV pathogenicity in pigs. CSFV infection can lead to an acute, subacute, chronic, or asymptomatic disease in pigs depending on CSFV strains’ virulence and other factors, such as the host’s age and immune status [15]. Although CSFV strains with different virulence exist globally, more and more reports show that the currently circulating strains are mainly moderately virulent [16,17,18,19].

Currently, the World Organization for Animal Health (OIE) lists 38 CSF-free members, which mainly locate in North America, part of South America, Oceania, and a large part of the European Union. CSF is still endemic in Asia, South and Central America, Eastern Europe, and parts of Africa. Additionally, specific zones in Brazil, Colombia, and Ecuador are declared CSF-free by OIE, while the other zones in these countries remain CSF-endemic [20]. Additionally, there is a risk of CSF re-emergence in CSF-free areas due to the existence of CSF-endemic regions in the world, as well as the reservoir of CSFV in wild boars. For instance, in 2018, CSF re-emerged in Japan after 26 years of CSF-free status, affecting both domestic pigs and wild boars [12]. Thus, as an endemic and re-emerging virus in pigs, CSFV is still a threat to the world’s pig industry.

Biosecurity measures are crucial for preventing and controlling CSF. According to the guidance of OIE, strict sanitary prophylaxis is the first barrier to prevent CSF outbreaks [21]. In brief, some key points could be employed, such as maintaining effective communication between veterinary authorities, veterinary practitioners, and pig farmers, establishing a reliable disease reporting system, implementing strict quarantine, and enhancing hygiene measures to prevent contact between domestic pigs and wild boars [21]. Laboratory diagnostic techniques are also needed. Rapid and sensitive detection methods for CSFV infection, such as reverse transcription-polymerase chain reaction (RT-PCR) [22], SYBR Green or TaqMan real-time RT-PCR [23,24], and reverse transcription loop-mediated isothermal amplification (RT-LAMP) [25], are crucial to informing the appropriate control measures. Additionally, due to that wild boars are susceptible to CSFV, eradication of CSF from wild boars is of epidemiologic value, which can prevent the spread of CSFV from wild boars to domestic pigs [2]. If CSF outbreaks occur, strict measures must be implemented to curb the epidemic. For example, suspected and infected pig herds must be slaughtered and animal carcasses should be buried or incinerated; thorough disinfection is also needed; infected zones must be designated and then pig movements should be restricted [21].

Currently, prophylactic vaccination is still the primary strategy for preventing and controlling CSF in CSF-endemic regions. Traditional CSF live attenuated vaccines (LAVs) strains, such as C-strain, LPC strain, LK-VNIVViM strain, GPE-strain, Thiverval strain, and PAV-250 strain, could provide an effective, rapid, and solid immune protection against CSFV infection and have been widely used to fight CSF [26]. Although traditional LAVs are effective and safe, they lack a serological concept of differentiating infected from vaccinated animals (DIVA). They thus are not conducive to CSF eradication in CSF-endemic areas. Therefore, it is necessary to develop CSF marker vaccines with DIVA capabilities. Currently, many efforts have been performed to develop novel CSF marker vaccines. Holinka et al. [27] reported that FlagT4Gv, a live attenuated marker vaccine, could induce protection against challenge with virulent CSFV as early as three days post-vaccination and an increase of IFN-α three days after FlagT4Gv vaccination might play an important role in the immune protection, which meant that a novel CSF LAV with DIVA capabilities was a good choice for CSF control. Additionally, in recent years, novel subunit marker vaccines based on the CSFV E2 protein have been developed for alternative options against CSF [26]. For example, a commercial CSF marker vaccine TWJ-E2^®^ (Tecon Bio-technology Co., Ltd., Urumqi, China), containing a baculovirus-expressed E2 glycoprotein of vaccine C-strain of genotype 1.1, was reported to provide complete protection to pigs against lethal challenge with virulent Shimen strain of genotype 1.1 and heterologous strains of genotype 2 [28,29].

Despite decades of efforts, it is still difficult to eliminate CSF in endemically affected regions and re-emerging areas [30,31]. The emergence of moderate or attenuated CSFV strains results in persistent recessive infection and immunosuppression in pigs, making it more challenging to control CSF [32,33]. Thus, in addition to implementing enhanced biosecurity measures and exploring more effective CSF vaccines, other strategies are also needed for effectively controlling CSF. Currently, more and more research about anti-CSFV strategies was carried out by scientists, because of the great prospects and value of anti-CSFV strategies in the prevention and control of CSF. In this article, we aim to introduce the research progress of anti-CSFV strategies. In detail, host proteins with anti-CSFV effects, host proteins whose function defect exert anti-CSFV effects, drug candidates with anti-CSFV effects, and RNA interference (RNAi) targeting CSFV viral genes were mentioned and the possible mechanisms related to anti-CSFV effects were summarized.

## 2. Host Proteins with Anti-CSFV Effects

The interaction between virus and host exists in the process of virus infection. After viral infection, the host will quickly initiate the innate immune response to achieve the goal of eliminating the virus. Previous studies have demonstrated that many host proteins had anti-CSFV activity (Table 1). In general, the over-expression or functional activation of this kind of protein inhibits CSFV replication, whereas protein knockdown or functional inhibition enhances CSFV replication. According to the possible mechanisms associated with the anti-CSFV effects, these host proteins could be divided into the following categories. (1) Once viral infection occurs, the type I interferon (IFN) pathway might be triggered, resulting in the expression of hundreds of interferon-stimulated genes (ISGs). Proteins encoded by ISGs, such as ISG15 [34], Viperin (RSAD2) [35,36], NRAMP1, NT5C3A, CXCL10, OAS1 [37], Mx (porcine Mx1, porcine Mx2, human MxA, and mouse Mx1) [38,39,40], GBP1 [41], pOASL [42], and IFITM family (IFITM1, IFITM2, and IFITM3) [43], have been shown to have anti-CSFV effects. Additionally, ISGs have been further used in the research of transgenic pigs resistant to CSFV. It was reported that cells from transgenic pigs over-expressing Mx or RSAD2 gene had an antiviral capacity against CSFV [44,45,46]. (2) Some proteins can inhibit CSFV replication by regulating the NF-κB signaling pathway, such as TRAF6 [47], Trx2 [48], and Hsp27 [49]. (3) Some proteins can inhibit virus replication by affecting the activity of the RIG-I-like signaling pathway, such as HB [50] and MAVS [51]. (4) Proteins involved in the ubiquitin-proteasome system, such as pRNF114 [52] and PSMB10 [53], could also inhibit CSFV replication. (5) Some proteins inhibit CSFV replication by modulating the Toll-like receptors (TLRs) pathway, such as uS10 [54]. (6) Other proteins also could inhibit CSFV replication. For example, SERINC5, a host-restricted cytokine, inhibits CSFV replication via activation of MDA5-mediated type I IFN signaling pathway [55]; LDHB decreases CSFV replication possibly related to the regulation of mitophagy [56]; The eukaryotic elongation factor 1A, eEF1A, also suppress the CSFV growth markedly [57].

Since host proteins mentioned above can inhibit CSFV replication, it is valuable to develop drugs that can activate the functions of these anti-CSFV proteins. Theoretically, these drug candidates can also exert antiviral effects. Additionally, these antiviral proteins can be used to prepare transgenic pigs. Pigs that over-express antiviral genes can theoretically resist or reduce CSFV infection.

## 3. Host Proteins Whose Function Defect Exert Anti-CSFV Effects

Different host proteins might play the opposite role in the process of viral infection. In addition to the host proteins with antiviral effects mentioned above, there is another kind of host protein, which is utilized or hijacked by the virus and might promote virus replication. Previous studies have revealed that some host proteins played an important role in the process of CSFV infection or replication and are required for the life cycle of CSFV (Table 2). They have the potential to be anti-CSFV targets, due to that the function defect of this kind of host protein could inhibit CSFV replication. These host proteins could be divided into the following categories according to their possible mechanism involved in CSFV replication. (1) Many members of the small Rab GTPase family, such as Rab1A [58], Rab1b [59], Rab2 [60], Rab5 [61,62], Rab7, Rab11 [62], Rab18 [63], and Rab25 [64], play a critical role in CSFV replication. For example, Rab18 interacts with CSFV NS5A and mediates viral RNA replication and virion assembly [63]. (2) Some proteins could promote CSFV replication possibly through regulating the level of interferons (IFNs), such as PKR [65], PCBP1 [66], and MERTK [67]. (3) Some host proteins can help CSFV enter cells. For example, Tsg101 participates in clathrin-mediated endocytosis of CSFV [68]; LamR is a cellular attachment receptor for CSFV [69]; Anx2 is a cellular membrane protein likely associated with CSFV entry [70]; Integrin β3 is membrane-bound signal mediator related to the CSFV infection [71]. Some members of the small Rab GTPase family are also involved in virus entry; Rab5, Rab7, and Rab11 are needed for caveola-dependent endocytosis of CSFV in porcine alveolar macrophages [62]. (4) Some host proteins affect both CSFV replication and virulence through interacting with viral proteins, such as CCDC115 [72], SERTAD1 [73], DCTN6 [74], and IQGAP1 [75], which have been proved by both in vivo and in vitro studies. (5) Some host proteins or enzymes are beneficial for viral RNA replication, translation, and assembly, such as RHA [76] and eIF3E [77]. (6) Some proteins related to the function of Golgi and/or endoplasmic reticulum (ER), such as GBF1 [60], OS9 [78], and GRP78 [79], are important for CSFV replication. (7) Proteins related to MAPK signaling pathways, such as TRAF5 [80] and MEK2 [81,82], could promote CSFV replication. (8) Some proteins involved in autophagy or apoptosis, such as BECN1, LC3 [83], NDP52 [84], and FHC [85], also could regulate CSFV replication. (9) Other proteins were also reported to be involved in the CSFV replication, such as FKBP8 [86], Jiv90 [87], HSP70 [88], HO-1 [89], and AIF1 [90].

Since these proteins are potential anti-CSFV targets, scientists can consider developing some molecules that inhibit the functions of these proteins. Theoretically, these drug candidates can inhibit CSFV replication and have potential value in CSF treatment. Importantly, while verifying the antiviral effects of these drug candidates, it is necessary to test whether these drug candidates affect cell viability and/or have an adverse effect on pigs. Since the host proteins, required for CSFV infection or replication, might also play an important role in the cell life. While destroying the functions of these proteins exerts antiviral effects, the normal functions of cells or the body might also be disrupted.

Additionally, according to possible mechanism by which host proteins affect CSFV replication (Table 1 and Table 2), we found that most of host proteins reported in the literature regulated the CSFV replication by interacting with CSFV viral proteins, such as interaction of Mx1 and CSFV NS5B [38], interaction of PCBP1 and CSFV N^pro^ [66], and interaction of Anx2 and CSFV E2 [70], which meant that the interaction between host protein and viral protein played an important role in CSFV replication. High-throughput screening methods, such as yeast two-hybrid (Y2H) assay, have been utilized to screen the host proteins potentially interacting with CSFV viral proteins [47,48,54,66,91,92,93]. However, only a small number of host proteins have been further confirmed to interact with CSFV viral proteins and affect CSFV replication. Thus, it is necessary to use co-immunoprecipitation, glutathione *S*-transferase pulldown, laser confocal microscopy or other methods to further verify the potential interaction obtained by high-throughput screening and then to evaluate the impact of interactions on CSFV replication. We believe that studies on the interaction between host protein and CSFV viral protein will be conducive to the discovery of novel antiviral proteins or antiviral targets.

## 4. Drug Candidates with Anti-CSFV Effects

Many efforts have been made for developing anti-CSFV drugs. CSFV life cycle mainly includes virus attachment, receptor binding and virus entry, virus uncoating, viral RNA replication, translation and processing of viral proteins, virion morphogenesis, and virus release [95]. It was reported that some molecules could target the CSFV life cycle, thereby exerting antiviral effects (Table 3). (1) Targeting the viral polymerase is an effective strategy against viruses. CSFV NS5B is an RNA-dependent RNA polymerase (RdRp), which is a key enzyme initiating viral RNA replication. It was reported that viral polymerase inhibitors, such as BPIP [96,97,98], VP32947 [99], and BBP/CSFA-0 and its analogues [100], could inhibit CSFV replication in vivo and/or in vitro. Among them, the anti-CSFV effects of BPIP have been deeply studied; this drug could inhibit CSFV replication in vitro by targeting the viral polymerase, reduce CSFV infection in pigs and also reduce CSFV transmission to untreated pigs [96,97,98]. (2) Some recombinant proteins with ribonuclease (RNase) activity could suppress viral replication through degrading viral RNA. The recombinant antibody with RNA-hydrolyzing activity (3D8 scFv) [101] and *Staphylococcus aureus* nuclease fused with CSFV capsid protein (Cap-SNase) [102] could suppress CSFV propagation possibly by targeting viral RNA. (3) CSFV glycoproteins (E^rns^, E1, and E2) are located on the external part of viral particles. These glycoproteins form heterodimeric and/or homodimeric complexes, which are important for the stability of viral glycoproteins and the ability of viral infection. Some molecules targeting the glycosylation process, such as GP6 [103], glycosylation inhibitors (tunicamycin, IW3, and IW7) [104,105], and analogs of glycosyltransferase substrates [106,107], could inhibit the formation of glycoprotein complexes and virus yield.

Moreover, when studying the mechanism of CSFV replication, scientists found that many intracellular signaling pathways or biological processes were involved in the process of CSFV replication and many molecules had anti-CSFV activity by targeting these intracellular signaling pathways or biological processes (Table 3). (1) Both IFNs and IFN-stimulated genes (ISGs) are important for the host to resist viral infection. IFN-α/γ [34,108,109] and recombinant protein encoded by ISGs, such as porcine Mx1 fused to HIV Tat protein transduction domain (PTD-poMx1) [40,110], have anti-CSFV effects. The effect of IFN-α treatment on CSFV infection in swine was reported for the first time by Fernandez-Sainz et al. [109]. They used the replication defective recombinant human adenovirus type 5 expressing porcine IFN-α (Ad5-pIFNα) to pretreat swine experimentally infected with highly virulent CSFV. Although the pretreatment with Ad5-pIFNα could not prevent lethal disease, it indeed delayed the appearance of CSF-related clinical signs and viral replication [109], which suggested that IFN-α was a potential anti-CSFV agent. Additionally, Zhang et al. [110] reported that treatment with PTD-poMx1 alleviated CSF-related symptoms and viral load in CSFV-infected pigs, but could not completely block CSFV replication, which meant that PTD-poMx1 could provide partial protection against CSFV challenge. (2) Intracellular cholesterol and its transport play an important role in CSFV infection and replication [111,112]. Drug reducing cellular cholesterol levels (MβCD and 25-hydroxycholesterol) [111], or inhibiting cholesterol transport (U18666A and imipramine) [112], could inhibit CSFV replication. (3) Previous studies have demonstrated CSFV infection could trigger a functional autophagy pathway, which was important for CSFV replication and release in host cells [83,113]. The induction of autophagy with rapamycin increases virus yield, while inhibiting the autophagy with 3-MA decreases virus yield [83]. (4) Free fatty acids are required for CSFV replication; inhibitors of fatty acid biosynthesis (C75 and TOFA) or inhibitors of fatty acid beta-oxidation (etomoxir and TMZ) [114] affect virus production. (5) CSFV could utilize intracellular membrane organelles for its replication. Drug regulating the function of Golgi and/or ER, such as inhibitors of vesicular transport between Golgi and ER (BFA, CI-976, and GCA) [59,60], ER stress inhibitors (TUDCA and 4-PBA) [115], and IRE1 endonuclease inhibitor (4μ8c) [116], significantly inhibit CSFV replication. (6) TLRs signaling pathways are involved in CSFV replication; TLR-specific ligands, such as LPS [117,118] and R837 [118], exert inhibitory effects on CSFV replication. (7) Drugs targeting the MAPK signaling pathway, such as SB203580 (an inhibitor of p38 MAPK activation) [80] and U0126 (specific inhibitor for MEK1/2/ERK1/2) [82], could suppress CSFV replication. (8) The proteasome is involved in the interplay between many viruses and hosts. MG132 [119], a proteasome inhibitor, could attenuate the CSFV replication. (9) Other molecules also have anti-CSFV effects, such as Prostaglandin A1 [120,121], the phage-displayed E2-binding peptides [122], ceramide (C6) (activator of the protein phosphatase 1 pathway) [123], and quercetin (inhibitor of HSP70 function) [88]. More and more anti-CSFV drug candidates will be discovered with the increase of antiviral research.

## 5. RNA Interference (RNAi) Targeting Viral Genes

RNA interference (RNAi) is an intracellular mechanism for post-transcriptional gene silence induced by small interfering RNAs (siRNAs) of about 21–23 nt, which are homologous to the mRNA of target genes [124]. RNAi has been successfully applied to inhibit the replication of many viruses. The application of RNAi strategy for controlling CSF is also promising. Studies have shown that RNAi targeting CSFV viral genes, such as N^pro^, p7, NS3, NS4A, NS5A, NS5B, and C [125,126,127,128,129,130], had anti-CSFV effects. Li et al. [127] revealed that RNAi targeting single or multiple viral genes could efficiently inhibit CSFV replication and the anti-CSFV effect was markedly stronger when interfering with multiple viral genes. Moreover, both short hairpin RNAs (shRNAs) and siRNAs technology are effective RNAi strategies for targeting CSFV viral genes. Importantly, RNAi strategy targeting CSFV viral genes is also valuable for research of transgenic pigs resistant to CSFV [131,132]. The transgenic pigs expressing anti-CSFV shRNAs could effectively limit the CSFV replication and reduce CSFV-associated clinical signs [131].

## 6. Concluding Remarks and Prospects

**Figure 2:**
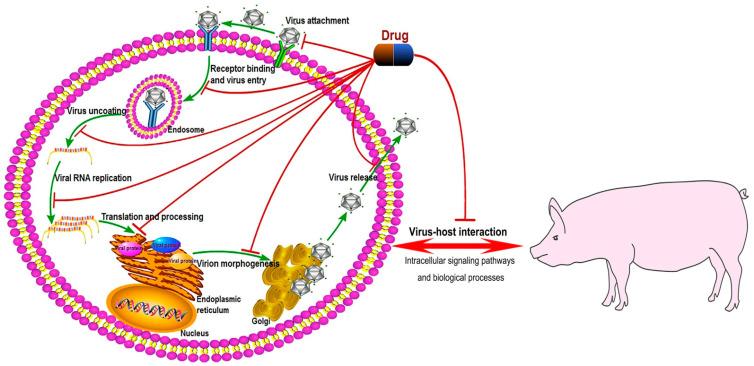
Anti-CSFV drug candidates target the CSFV life cycle or virus-host interaction. The schematic diagram of the CSFV life cycle in Figure 2 referred to the previous literature reported by Li et al. [95] with some modifications. Pathway Builder Tool 2.0 was used to draw the schematic diagram of the CSFV life cycle. Anti-CSFV molecules could target the CSFV life cycle including virus attachment, receptor binding and virus entry, virus uncoating, viral RNA replication, translation and processing of viral proteins, virion morphogenesis, and virus release. Anti-CSFV molecules could also target the intracellular signaling pathways or biological processes related to CSFV infection and replication, such as type I IFN signaling pathway, NF-κB signaling pathway, RIG-I-like signaling pathway, ubiquitin-proteasome system, TLRs signaling pathway, MAPK signaling pathways, autophagy, apoptosis, and metabolism and transport of lipids.

CSF is an ancient zoonotic disease, which has caused substantial economic losses to the pig industry worldwide. The control of this disease is still a major problem for the pig industry. Currently, it is difficult to completely eradicate CSF in CSF-endemic and re-emerging areas through biosecurity and vaccination strategies. Therefore, it is meaningful to develop anti-CSFV drugs or other strategies. In this review, we summarized the anti-CSFV strategies into several aspects including host proteins with anti-CSFV effects, host proteins whose function defect exert anti-CSFV effects, drug candidates with anti-CSFV effects, and RNAi targeting CSFV viral genes. Many host proteins and chemical molecules exert anti-CSFV effects by targeting the process of virus replication including viral attachment and entry, viral genome replication, translation and post-translational modification of viral proteins, and the assembly of the viral particle (Figure 2). Furthermore, many intracellular signaling pathways or biological processes have been shown to be related to CSFV infection and replication, such as type I IFN signaling pathway, NF-κB signaling pathway, RIG-I-like signaling pathway, ubiquitin-proteasome system, TLRs signaling pathway, MAPK signaling pathways, autophagy, apoptosis, and metabolism and transport of lipids. These pathways or biological processes can be utilized for the development of anti-CSFV targets or drugs (Figure 2). Although the commercial and specific anti-CSFV drug is still not available, the increasing antiviral research will help the discovery of anti-CSFV drugs. Additionally, due to that members of the Flaviviridae have similar genomic structures and replication strategies [133], studies on anti-CSFV strategies could be used as a reference for other viruses in the Flaviviridae family, such as hepatitis C virus (HCV), dengue virus, and Zika virus. For example, previous studies have demonstrated that some molecules, such as analogs of glycosyltransferase substrates [106,107], could inhibit the replication of both CSFV and HCV.

After summarizing and analyzing the results of previous anti-CSFV research, we make some suggestions, which might benefit further anti-CSFV research. (1) Animal experiments are needed for the evaluation of the effect of anti-CSFV strategies. We found that cells were selected as the main object for the anti-CSFV research in previous reports, and animal experiments were rarely involved. Thus, we recommend that researchers could use animals to evaluate the effects of antiviral strategies after antiviral strategies have achieved good antiviral effects at the cellular level. (2) It is necessary to evaluate the safety of antiviral strategies to animals, because some antiviral strategies may be toxic to animals. For example, Dai et al. [134] reported that an early lethality due to anti-CSFV shRNA was observed in shRNA-transgenic pigs; they revealed that shRNA caused adverse effects and shRNA-induced disruption of the endogenous miRNA pathway might lead to the early lethality of shRNA-transgenic pigs. (3) The issue of drug resistance needs attention. Previous studies have demonstrated that drug-resistant CSFV strains could be induced in vitro through the serial passage of the virus in increasing drug-concentration [96,100], which meant that clinical drug-resistant CSFV strains might also emerge if the drug was used in the clinic. (4) MiRNAs could be used to develop new potential antiviral strategies. Previous studies have shown that miRNAs play an important role during viral infection [135,136]. MiRNAs can inhibit or promote the replication of viruses, such as dengue virus [135] and HCV [136], which shows that miRNA mimics and miRNA inhibitors are potential antiviral drug candidates. MiRNAs could also regulate CSFV replication. For example, miR-140 inhibits CSFV replication by targeting Rab25 [64]. However, there are still few studies on the relationship between miRNA and CSFV replication. Further research is needed for discovering miRNAs that affect CSFV replication. (5) Previous studies have revealed that in vitro screening of chemical libraries facilitated the acquisition of potential inhibitors of viral replication, such as SARS-CoV-2 [137]. Thus, we think commercial antiviral compound libraries can be used to screen anti-CSFV drugs, which has not yet been reported. (6) Studies on the pathogenic mechanism of CSF will facilitate the discovery of antiviral targets or drugs. Although many studies related to the mechanism of CSFV replication have been reported, the pathogenesis of CSF is still poorly understood. For effective prevention and control of CSF, it is necessary to conduct in-depth and systematic research on the pathogenesis of CSF.

If effective anti-CSFV drugs, targeting the CSFV life cycle or CSFV-host interaction, are developed, approved, and commercialized, they will be beneficial to the prevention and control of CSF, especially during the outbreak of CSF. The use of anti-CSFV drugs in combination with vaccination will be an effective emergency preventive measure against CSF for pigs in infected zone during CSF outbreak. Notably, the selected anti-CSFV drugs need to be proven not to affect the immune effect of vaccines. The use of anti-CSFV drugs might establish a robust antiviral state in pig herd and inhibit CSFV replication and spread [109], which will allow enough time for vaccines to exert immunoprotective effects. 

## Figures and Tables

**Table 1:** Host proteins with anti-classical swine fever virus (CSFV) effects.

No.	Host Protein	Possible Mechanisms Associated with Anti-CSFV Effects of the Host Protein	Ref.
1.	ISG15	Inhibit CSFV replication via inhibition of autophagy by ISGylating BECN1.	[34]
2.	Viperin *	Interact with CSFV E2 and NS5A; its anti-CSFV function occurs during the viral genome and/or protein synthesis step.	[35,36]
3.	NRAMP1, NT5C3A, CXCL10, OAS1	Interferon-stimulated genes (ISGs); inhibit CSFV replication.	[37]
4.	Mx * (porcine Mx1, porcine Mx2, human MxA, and mouse Mx1)	IFN-induced GTPases; inhibit CSFV replication; porcine Mx1 interacts with CSFV NS5B and undermines the RNA-dependent RNA polymerase (RdRp) activities of NS5B.	[38,39,40]
5.	GBP1	IFN-induced GTPase; interact with CSFV NS5A; act mainly on the early phase of CSFV replication.	[41]
6.	pOASL	Inhibit CSFV replication via the MDA5-mediated type I IFN signaling pathway.	[42]
7.	IFITM family (IFITM1, IFITM2, and IFITM3)	IFN-inducible transmembrane proteins; IFITM1, IFITM2, and IFITM3 colocalization with Lamp1, IFITM2 with Rab5 and Rab7, and IFITM3 with Rab7 appear in CSFV-infected cells.	[43]
8.	TRAF6	Interact with NS3 and inhibit CSFV replication via activation of NF-κB signaling pathway along with the increase in expression of IFN-β and IL-6.	[47]
9.	Trx2	Interact with CSFV E2 and inhibit CSFV replication via NF-κB signaling pathway.	[48]
10.	Hsp27	Interact with CSFV NS5A and inhibit CSFV replication by NF-κB signaling pathway.	[49]
11.	HB	Interact with CSFV capsid (C) protein; antagonize CSFV replication by regulating RIG-I pathway and IFN pathway.	[50]
12.	MAVS	The adaptor of the RIG-I-like receptor; induce antiviral cytokines and apoptosis to inhibit CSFV replication.	[51]
13.	pRNF114	RING domain E3 ubiquitin ligase; interact with the CSFV NS4B and mediate the K27-linked polyubiquitination and degradation of NS4B through a proteasome-dependent pathway.	[52]
14.	PSMB10	Interact with CSFV NS3 and mediate the degradation of NS3 through the ubiquitin-proteasome system; restore the function of MHC class I antigen presentation and inhibit CSFV proliferation.	[53]
15.	uS10	Interact with CSFV N^pro^ and inhibit CSFV replication by modulating TLR3 expression.	[54]
16.	SERINC5	Inhibit CSFV replication via activation of MDA5-mediated type I IFN signaling pathway.	[55]
17.	LDHB	Interact with CSFV NS3 and decrease CSFV replication possibly related to the regulation of mitophagy.	[56]
18.	eEF1A	Interact with the CSFV NS5A; bind to the CSFV IRES; reduce the translation efficiency of CSFV IRES.	[57]

* Evidence regarding to host proteins affecting CSFV replication was supported from animal studies.

**Table 2:** Host proteins whose function defect can exert anti-CSFV effects.

No.	Host Protein	Possible Mechanism by Which Host Proteins Influence CSFV Replication	Ref.
1.	Rab1A	Be required for viral particle assembly; bind to viral particle assembly-related NS5A protein.	[58]
2.	Rab1b	Rab1b-GBF1-ARFs mediated intracellular trafficking is required for CSFV replication.	[59]
3.	Rab2	Be involved in Golgi function and promote CSFV proliferation.	[60]
4.	Rab5	Enhance CSFV proliferation and interact with CSFV NS4B to facilitate the formation of NS4B-related complex.	[61]
5.	Rab5, Rab7, and Rab11	Rab5 and Rab7 are required for clathrin-mediated endocytic pathway of CSFV in porcine kidney cells; Rab5, Rab7, and Rab11 are needed for caveola-dependent endocytosis of CSFV in porcine alveolar macrophages.	[62,94]
6.	Rab18	Interact with CSFV NS5A and mediate viral RNA replication and virion assembly.	[63]
7.	Rab25	Promote CSFV replication.	[64]
8.	PKR	PKR activation enhances CSFV replication; inhibition of PKR results in the reduction of CSFV replication and an increase in IFN induction.	[65]
9.	PCBP1	Interact with CSFV N^pro^ and promote CSFV growth by downregulating type I IFN.	[66]
10.	MERTK	Interact with CSFV E2 and facilitate virus entry; after virus entry, down-regulate IFN-β and promote CSFV infection.	[67]
11.	Tsg101	Participate in clathrin-mediated endocytosis of CSFV and regulate the viral replication process by interacting with CSFV NS4B and NS5B.	[68]
12.	LamR	A cellular attachment receptor for CSFV; interact with the CSFV E^rns^.	[69]
13.	Anx2	Cellular membrane protein likely associated with CSFV entry into cells; interact with CSFV E2 and promote CSFV multiplication.	[70]
14.	integrin β3	Membrane-bound signal mediator; be required in CSFV infection and proliferation.	[71]
15.	CCDC115 *	Interaction of CCDC115 with CSFV E2 plays an important role in virus replication and virulence.	[72]
16.	SERTAD1 *	Interaction of SERTAD1 with CSFV E2 plays a critical role in virus replication and virulence.	[73]
17.	DCTN6 *	Interaction of DCTN6 with CSFV E2 plays a role in virus replication and virulence.	[74]
18.	IQGAP1 *	Interaction between IQGAP1 and CSFV C protein is essential for virus replication and virulence.	[75]
19.	RHA	RNA helicase; bind the 5′-UTR and 3′-UTR of CSFV; be involved in the expression and replication of CSFV.	[76]
20.	eIF3E	The component of eukaryotic translation initiation factor; interact with CSFV NS5A; enhance the translational activity of CSFV IRES.	[77]
21.	GBF1	Be involved in Golgi function and promote CSFV proliferation.	[60]
22.	OS9	Be involved in the endoplasmic reticulum-associated degradation pathway; interaction of OS9 with CSFV C protein is involved in CSFV replication.	[78]
23.	GRP78	The monitor of unfolded protein response (UPR) signaling pathways; interact with CSFV NS5A and enhance viral replication.	[79]
24.	TRAF5	Interact with CSFV NS3 and promote CSFV replication via p38 MAPK activation.	[80]
25.	MEK2	Interact with CSFV E2 and promote CSFV growth via attenuation of the JAK-STAT signaling pathway.	[81,82]
26.	BECN1, LC3	Be involved in cellular autophagy; affect progeny virus production.	[83]
27.	NDP52	An autophagy receptor; mediate CSFV infection.	[84]
28.	FHC	Interact with CSFV NS4B, enhance CSFV replication and play a positive role in viral anti-apoptosis by regulating ROS accumulation.	[85]
29.	FKBP8	Interact with CSFV NS5A and promote viral RNA replication.	[86]
30.	Jiv90	Molecular chaperone; promote viral RNA replication.	[87]
31.	HSP70	Interact with CSFV NS5A and promote viral RNA synthesis.	[88]
32.	HO-1	Down-regulation of HO-1 inhibits CSFV proliferation.	[89]
33.	AIF1	Promote CSFV replication and IL-6 production.	[90]

* Evidence regarding to host proteins affecting CSFV replication was supported from animal studies.

**Table 3:** Drug candidates with anti-classical swine fever virus (CSFV) activity.

No.	Drug Candidates	Possible Mechanism Related to the Antiviral Effect of Drug Candidates	Ref.
1.	BPIP *	A viral polymerase inhibitor; inhibit CSFV replication by targeting the viral polymerase.	[96,97,98]
2.	VP32947	A small molecule inhibitor of pestivirus replication; possibly target RNA-dependent RNA polymerase.	[99]
3.	BBP/CSFA-0 and its analogues	Pestivirus inhibitors; target the RNA-dependent RNA polymerase.	[100]
4.	3D8 scFv	A recombinant antibody with RNA-hydrolyzing and cell-penetrating activities; suppress CSFV replication possibly by targeting viral RNA genomes or transcripts.	[101]
5.	Cap-SNase	The fusion protein of CSFV capsid (Cap) and *Staphylococcus aureus* nuclease (SNase); inhibit the production of CSFV based on the capsid-targeted viral inactivation.	[102]
6.	GP6	A novel glycosyl sulfoxide; probably target the late steps of the glycosylation process of CSFV E2 and E^rns^.	[103]
7.	tunicamycin, IW3, IW7	Inhibitor of glycosylation; inhibit N-glycosylation process of CSFV glycoproteins.	[104,105]
8.	analogs of glycosyltransferase substrates	Inhibit CSFV replication possibly related to the glycosylation process of viral proteins.	[106,107]
9.	IFN-α *, IFN-γ	IFNs induce the expression of interferon-stimulated genes (ISGs) for defense against viral infection.	[34,108,109]
10.	PTD-poMx1 *	Porcine Mx1 fused to HIV Tat protein transduction domain (PTD); inhibit CSFV replication in vitro and in vivo via the antiviral activity of Mx1 protein.	[40,110]
11.	MβCD, 25-hydroxycholesterol	Regulate the level of cellular cholesterol and inhibit CSFV replication.	[111]
12.	U18666A, imipramine	Inhibitor of cholesterol transport; disrupt cholesterol trafficking and then affect CSFV replication.	[112]
13.	3-MA	Inhibit autophagy; decrease virus yield.	[83]
14.	C75, TOFA	Inhibitors of fatty acid biosynthesis; inhibit CSFV replication.	[114]
15.	etomoxir, TMZ	Inhibitors of fatty acid beta-oxidation; inhibit CSFV replication.	[114]
16.	BFA, GCA, and CI-976	Inhibitors of vesicular transport between Golgi and ER; inhibit CSFV infection.	[59,60]
17.	4-PBA, TUDCA	ER stress inhibitors; inhibit CSFV replication.	[115]
18.	4μ8c	A specific IRE1 endonuclease inhibitor; block the IRE1-XBP1 signal related to unfolded protein response (UPR); reduce CSFV replication.	[116]
19.	LPS	Induce mRNA of IFN-α and IFN-β; impair CSFV replication possibly related to PKR activation.	[117]
20.	LPS-B5, R837	Lipopolysaccharide from *Escherichia coli* 055:B5 (LPS-B5) and imiquimod (R837); TLR-specific ligands; exert inhibitory effects on CSFV replication.	[118]
21.	SB203580	Inhibitor of p38 MAPK activation; suppress CSFV replication.	[80]
22.	U0126	A specific inhibitor for MEK1/2/ERK1/2; inhibit CSFV replication.	[82]
23.	MG132	A proteasome inhibitor; inhibit CSFV replication possibly via the activation of the JAK-STAT pathway and the up-regulation of ISGs expression.	[119]
24.	PGA1	Prostaglandin A1; inhibit CSFV replication.	[120,121]
25.	phage-displayed E2-binding peptides	CSFV-specific ligands; phage displaying the octapeptide sequence DRATSSNA; inhibit CSFV replication possibly through binding CSFV E2 protein.	[122]
26.	ceramide (C6)	Activator of the protein phosphatase 1 (PP1) pathway; inhibit CSFV replication via activation of the PP1 pathway.	[123]
27.	Quercetin	Inhibit the function of HSP70; decrease CSFV replication.	[88]

* Evidence regarding to drug candidates with anti-CSFV activity was supported from animal studies.

## Data Availability

Data sharing is not applicable.

## References

[1] Paton D.J., Greiser-Wilke I. (2003). Classical swine fever—An update. Res. Vet. Sci..

[2] Moennig V. (2015). The control of classical swine fever in wild boar. Front. Microbiol..

[3] Thiel H.J., Stark R., Weiland E., Rümenapf T., Meyers G. (1991). Hog cholera virus: Molecular composition of virions from a pestivirus. J. Virol..

[4] Beer M., Reimann I., Hoffmann B., Depner K. (2007). Novel marker vaccines against classical swine fever. Vaccine.

[5] Paton D.J., McGoldrick A., Greiser-Wilke I., Parchariyanon S., Song J.Y., Liou P.P., Stadejek T., Lowings J.P., Björklund H., Belák S. (2000). Genetic typing of classical swine fever virus. Vet. Microbiol..

[6] Postel A., Schmeiser S., Perera C.L., Rodríguez L.J., Frias-Lepoureau M.T., Becher P. (2013). Classical swine fever virus isolates from Cuba form a new subgenotype 1.4. Vet. Microbiol..

[7] Deng M.C., Huang C.C., Huang T.S., Chang C.Y., Lin Y.J., Chien M.S., Jong M.H. (2005). Phylogenetic analysis of classical swine fever virus isolated from Taiwan. Vet. Microbiol..

[8] Pan C.H., Jong M.H., Huang T.S., Liu H.F., Lin S.Y., Lai S.S. (2005). Phylogenetic analysis of classical swine fever virus in Taiwan. Arch. Virol..

[9] Jiang D.L., Gong W.J., Li R.C., Liu G.H., Hu Y.F., Ge M., Wang S.Q., Yu X.L., Tu C. (2013). Phylogenetic analysis using E2 gene of classical swine fever virus reveals a new subgenotype in China. Infect. Genet. Evol..

[10] Gong W., Wu J., Lu Z., Zhang L., Qin S., Chen F., Peng Z., Wang Q., Ma L., Bai A. (2016). Genetic diversity of subgenotype 2.1 isolates of classical swine fever virus. Infect. Genet. Evol..

[11] Zhou B. (2019). Classical Swine Fever in China-An Update Minireview. Front. Vet. Sci..

[12] Postel A., Nishi T., Kameyama K.I., Meyer D., Suckstorff O., Fukai K., Becher P. (2019). Reemergence of Classical Swine Fever, Japan, 2018. Emerg. Infect. Dis..

[13] Blome S., Staubach C., Henke J., Carlson J., Beer M. (2017). Classical Swine Fever-An Updated Review. Viruses.

[14] Malik Y.S., Bhat S., Kumar O.R.V., Yadav A.K., Sircar S., Ansari M.I., Sarma D.K., Rajkhowa T.K., Ghosh S., Dhama K. (2020). Classical Swine Fever Virus Biology, Clinicopathology, Diagnosis, Vaccines and a Meta-Analysis of Prevalence: A Review from the Indian Perspective. Pathogens.

[15] Brown V.R., Bevins S.N. (2018). A Review of Classical Swine Fever Virus and Routes of Introduction into the United States and the Potential for Virus Establishment. Front. Vet. Sci..

[16] Hao G., Zhang H., Chen H., Qian P., Li X. (2020). Comparison of the Pathogenicity of Classical Swine Fever Virus Subgenotype 2.1c and 2.1d Strains from China. Pathogens.

[17] Choe S., Le V.P., Shin J., Kim J.H., Kim K.S., Song S., Cha R.M., Park G.N., Nguyen T.L., Hyun B.H. (2020). Pathogenicity and Genetic Characterization of Vietnamese Classical Swine Fever Virus: 2014–2018. Pathogens.

[18] Kameyama K.I., Nishi T., Yamada M., Masujin K., Morioka K., Kokuho T., Fukai K. (2019). Experimental infection of pigs with a classical swine fever virus isolated in Japan for the first time in 26 years. J. Vet. Med. Sci..

[19] Zhang H., Leng C., Tian Z., Liu C., Chen J., Bai Y., Li Z., Xiang L., Zhai H., Wang Q. (2018). Complete genomic characteristics and pathogenic analysis of the newly emerged classical swine fever virus in China. BMC Vet. Res..

[20] The World Organisation for Animal Health (OIE) Official Disease Status-Classical Swine Fever. https://www.oie.int/en/animal-health-in-the-world/official-disease-status/classical-swine-fever/.

[21] The World Organisation for Animal Health (OIE) Information on Aquatic and Terrestrial Animal Diseases-Classical Swine Fever (CSF). https://www.oie.int/en/animal-health-in-the-world/animal-diseases/Classical-swine-fever/.

[22] Pan C.H., Jong M.H., Huang Y.L., Huang T.S., Chao P.H., Lai S.S. (2008). Rapid detection and differentiation of wild-type and three attenuated lapinized vaccine strains of classical swine fever virus by reverse transcription polymerase chain reaction. J. Vet. Diagn. Investig..

[23] Pérez L.J., Díaz de Arce H., Tarradas J., Rosell R., Perera C.L., Muñoz M., Frías M.T., Nuñez J.I., Ganges L. (2011). Development and validation of a novel SYBR Green real-time RT-PCR assay for the detection of classical swine fever virus evaluated on different real-time PCR platforms. J. Virol. Methods.

[24] Hoffmann B., Beer M., Schelp C., Schirrmeier H., Depner K. (2005). Validation of a real-time RT-PCR assay for sensitive and specific detection of classical swine fever. J. Virol. Methods.

[25] Chen L., Fan X.Z., Wang Q., Xu L., Zhao Q.Z., Zhou Y.C., Liu J., Tang B., Zou X.Q. (2010). A novel RT-LAMP assay for rapid and simple detection of classical swine fever virus. Virol. Sin..

[26] Coronado L., Perera C.L., Rios L., Frías M.T., Pérez L.J. (2021). A Critical Review about Different Vaccines against Classical Swine Fever Virus and Their Repercussions in Endemic Regions. Vaccines.

[27] Holinka L.G., O’Donnell V., Risatti G.R., Azzinaro P., Arzt J., Stenfeldt C., Velazquez-Salinas L., Carlson J., Gladue D.P., Borca M.V. (2017). Early protection events in swine immunized with an experimental live attenuated classical swine fever marker vaccine, FlagT4G. PLoS ONE.

[28] Gong W., Li J., Wang Z., Sun J., Mi S., Xu J., Cao J., Hou Y., Wang D., Huo X. (2019). Commercial E2 subunit vaccine provides full protection to pigs against lethal challenge with 4 strains of classical swine fever virus genotype 2. Vet. Microbiol..

[29] Wang Z., Li J., Dou Z., Zheng K., Li S., Kou C., He S. (2018). Protective efficacy of the classical swine fever E2 subunit vaccine in experimentally infected pigs. Chin. J. Anim. Infect. Dis..

[30] Jang G., Kim J.A., Yoo H., Yang K., Yang H.S., Park C., Jeong K., Park C.K., Lyoo Y.S., Lee C. (2020). Genomic characterization of classical swine fever virus LOM variants with 3’-UTR INDELs from pigs on Jeju Island, South Korea. Arch. Virol..

[31] de Oliveira L.G., Gatto I.R.H., Mechler-Dreibi M.L., Almeida H.M.S., Sonálio K., Storino G.Y. (2020). Achievements and Challenges of Classical Swine Fever Eradication in Brazil. Viruses.

[32] Edwards S., Fukusho A., Lefèvre P.C., Lipowski A., Pejsak Z., Roehe P., Westergaard J. (2000). Classical swine fever: The global situation. Vet. Microbiol..

[33] Moennig V. (2000). Introduction to classical swine fever: Virus, disease and control policy. Vet. Microbiol..

[34] Li C., Wang Y., Zheng H., Dong W., Lv H., Lin J., Guo K., Zhang Y. (2020). Antiviral activity of ISG15 against classical swine fever virus replication in porcine alveolar macrophages via inhibition of autophagy by ISGylating BECN1. Vet. Res..

[35] Li W., Mao L., Cao Y., Zhou B., Yang L., Han L., Hao F., Lin T., Zhang W., Jiang J. (2017). Porcine Viperin protein inhibits the replication of classical swine fever virus (CSFV) in vitro. Virol. J..

[36] Xu C., Feng L., Chen P., Li A., Guo S., Jiao X., Zhang C., Zhao Y., Jin X., Zhong K. (2020). Viperin inhibits classical swine fever virus replication by interacting with viral nonstructural 5A protein. J. Med. Virol..

[37] Wang X., Li Y., Li L.F., Shen L., Zhang L., Yu J., Luo Y., Sun Y., Li S., Qiu H.J. (2016). RNA interference screening of interferon-stimulated genes with antiviral activities against classical swine fever virus using a reporter virus. Antivir. Res..

[38] Zhou J., Chen J., Zhang X.M., Gao Z.C., Liu C.C., Zhang Y.N., Hou J.X., Li Z.Y., Kan L., Li W.L. (2018). Porcine Mx1 Protein Inhibits Classical Swine Fever Virus Replication by Targeting Nonstructural Protein NS5B. J. Virol..

[39] Zhao Y., Pang D., Wang T., Yang X., Wu R., Ren L., Yuan T., Huang Y., Ouyang H. (2011). Human MxA protein inhibits the replication of classical swine fever virus. Virus Res..

[40] He D.N., Zhang X.M., Liu K., Pang R., Zhao J., Zhou B., Chen P.Y. (2014). In vitro inhibition of the replication of classical swine fever virus by porcine Mx1 protein. Antivir. Res..

[41] Li L.F., Yu J., Li Y., Wang J., Li S., Zhang L., Xia S.L., Yang Q., Wang X., Yu S. (2016). Guanylate-Binding Protein 1, an Interferon-Induced GTPase, Exerts an Antiviral Activity against Classical Swine Fever Virus Depending on Its GTPase Activity. J. Virol..

[42] Li L.F., Yu J., Zhang Y., Yang Q., Li Y., Zhang L., Wang J., Li S., Luo Y., Sun Y. (2017). Interferon-Inducible Oligoadenylate Synthetase-Like Protein Acts as an Antiviral Effector against Classical Swine Fever Virus via the MDA5-Mediated Type I Interferon-Signaling Pathway. J. Virol..

[43] Li C., Zheng H., Wang Y., Dong W., Liu Y., Zhang L., Zhang Y. (2019). Antiviral Role of IFITM Proteins in Classical Swine Fever Virus Infection. Viruses.

[44] Zhao Y., Wang T., Yao L., Liu B., Teng C., Ouyang H. (2016). Classical swine fever virus replicated poorly in cells from MxA transgenic pigs. BMC Vet. Res..

[45] Yan Q., Yang H., Yang D., Zhao B., Ouyang Z., Liu Z., Fan N., Ouyang H., Gu W., Lai L. (2014). Production of transgenic pigs over-expressing the antiviral gene Mx1. Cell Regen..

[46] Xie Z., Jiao H., Xiao H., Jiang Y., Liu Z., Qi C., Zhao D., Jiao S., Yu T., Tang X. (2020). Generation of pRSAD2 gene knock-in pig via CRISPR/Cas9 technology. Antivir. Res..

[47] Lv H., Dong W., Cao Z., Li X., Wang J., Qian G., Lv Q., Wang C., Guo K., Zhang Y. (2017). TRAF6 is a novel NS3-interacting protein that inhibits classical swine fever virus replication. Sci. Rep..

[48] Li S., Wang J., He W.R., Feng S., Li Y., Wang X., Liao Y., Qin H.Y., Li L.F., Dong H. (2015). Thioredoxin 2 Is a Novel E2-Interacting Protein That Inhibits the Replication of Classical Swine Fever Virus. J. Virol..

[49] Ling S., Luo M., Jiang S., Liu J., Ding C., Zhang Q., Guo H., Gong W., Tu C., Sun J. (2018). Cellular Hsp27 interacts with classical swine fever virus NS5A protein and negatively regulates viral replication by the NF-κB signaling pathway. Virology.

[50] Li D., Dong H., Li S., Munir M., Chen J., Luo Y., Sun Y., Liu L., Qiu H.J. (2013). Hemoglobin subunit beta interacts with the capsid protein and antagonizes the growth of classical swine fever virus. J. Virol..

[51] Dong W., Lv H., Li C., Liu Y., Wang C., Lin J., Wang Y., Qian G., Guo K., Zhang Y. (2018). MAVS induces a host cell defense to inhibit CSFV infection. Arch. Virol..

[52] Zhang Y., Zhang H., Zheng G.L., Yang Q., Yu S., Wang J., Li S., Li L.F., Qiu H.J. (2019). Porcine RING Finger Protein 114 Inhibits Classical Swine Fever Virus Replication via K27-Linked Polyubiquitination of Viral NS4B. J. Virol..

[53] Deng S., Yang C., Nie K., Fan S., Zhu M., Zhu J., Chen Y., Yuan J., Zhang J., Xu H. (2019). Host cell protein PSMB10 interacts with viral NS3 protein and inhibits the growth of classical swine fever virus. Virology.

[54] Lv H., Dong W., Qian G., Wang J., Li X., Cao Z., Lv Q., Wang C., Guo K., Zhang Y. (2017). uS10, a novel Npro-interacting protein, inhibits classical swine fever virus replication. J. Gen. Virol..

[55] Li W., Zhang Z., Zhang L., Li H., Fan S., Zhu E., Fan J., Li Z., Chen W., Yi L. (2020). Antiviral Role of Serine Incorporator 5 (SERINC5) Proteins in Classical Swine Fever Virus Infection. Front. Microbiol..

[56] Fan S., Wu K., Zhao M., Yuan J., Ma S., Zhu E., Chen Y., Ding H., Yi L., Chen J. (2020). LDHB inhibition induces mitophagy and facilitates the progression of CSFV infection. Autophagy.

[57] Li S., Feng S., Wang J.H., He W.R., Qin H.Y., Dong H., Li L.F., Yu S.X., Li Y., Qiu H.J. (2015). eEF1A Interacts with the NS5A Protein and Inhibits the Growth of Classical Swine Fever Virus. Viruses.

[58] Lin J., Wang C., Liang W., Zhang J., Zhang L., Lv H., Dong W., Zhang Y. (2018). Rab1A is required for assembly of classical swine fever virus particle. Virology.

[59] Zhang L., Wang T., Song M., Jin M., Liu S., Guo K., Zhang Y. (2020). Rab1b-GBF1-ARFs mediated intracellular trafficking is required for classical swine fever virus replication in swine umbilical vein endothelial cells. Vet. Microbiol..

[60] Liang W., Zheng M., Bao C., Zhang Y. (2017). CSFV proliferation is associated with GBF1 and Rab2. J. Biosci..

[61] Lin J., Wang C., Zhang L., Wang T., Zhang J., Liang W., Li C., Qian G., Ouyang Y., Guo K. (2017). Rab5 Enhances Classical Swine Fever Virus Proliferation and Interacts with Viral NS4B Protein to Facilitate Formation of NS4B Related Complex. Front. Microbiol..

[62] Zhang Y.N., Liu Y.Y., Xiao F.C., Liu C.C., Liang X.D., Chen J., Zhou J., Baloch A.S., Kan L., Zhou B. (2018). Rab5, Rab7, and Rab11 Are Required for Caveola-Dependent Endocytosis of Classical Swine Fever Virus in Porcine Alveolar Macrophages. J. Virol..

[63] Zhang L., Zhao D., Jin M., Song M., Liu S., Guo K., Zhang Y. (2020). Rab18 binds to classical swine fever virus NS5A and mediates viral replication and assembly in swine umbilical vein endothelial cells. Virulence.

[64] Xu P., Jia S., Wang K., Fan Z., Zheng H., Lv J., Jiang Y., Hou Y., Lou B., Zhou H. (2020). MiR-140 inhibits classical swine fever virus replication by targeting Rab25 in swine umbilical vein endothelial cells. Virulence.

[65] Liu W.J., Yang Y.T., Zhao M.Q., Dong X.Y., Gou H.C., Pei J.J., Chen J.D. (2015). PKR activation enhances replication of classical swine fever virus in PK-15 cells. Virus Res..

[66] Li D., Li S., Sun Y., Dong H., Li Y., Zhao B., Guo D., Weng C., Qiu H.J. (2013). Poly(C)-binding protein 1, a novel N(pro)-interacting protein involved in classical swine fever virus growth. J. Virol..

[67] Zheng G., Li L.F., Zhang Y., Qu L., Wang W., Li M., Yu S., Zhou M., Luo Y., Sun Y. (2020). MERTK is a host factor that promotes classical swine fever virus entry and antagonizes innate immune response in PK-15 cells. Emerg. Microbes Infect..

[68] Liu C.C., Liu Y.Y., Cheng Y., Zhang Y.N., Zhang J., Liang X.D., Gao Y., Chen H., Baloch A.S., Yang Q. (2020). The ESCRT-I Subunit Tsg101 Plays Novel Dual Roles in Entry and Replication of Classical Swine Fever Virus. J. Virol..

[69] Chen J., He W.R., Shen L., Dong H., Yu J., Wang X., Yu S., Li Y., Li S., Luo Y. (2015). The laminin receptor is a cellular attachment receptor for classical Swine Fever virus. J. Virol..

[70] Yang Z., Shi Z., Guo H., Qu H., Zhang Y., Tu C. (2015). Annexin 2 is a host protein binding to classical swine fever virus E2 glycoprotein and promoting viral growth in PK-15 cells. Virus Res..

[71] Li W., Wang G., Liang W., Kang K., Guo K., Zhang Y. (2014). Integrin β3 is required in infection and proliferation of classical swine fever virus. PLoS ONE.

[72] Vuono E.A., Ramirez-Medina E., Berggren K., Rai A., Pruitt S., Silva E., Velazquez-Salinas L., Gladue D.P., Borca M.V. (2020). Swine Host Protein Coiled-Coil Domain-Containing 115 (CCDC115) Interacts with Classical Swine Fever Virus Structural Glycoprotein E2 during Virus Replication. Viruses.

[73] Vuono E.A., Ramirez-Medina E., Azzinaro P., Berggren K.A., Rai A., Pruitt S., Silva E., Velazquez-Salinas L., Borca M.V., Gladue D.P. (2020). SERTA Domain Containing Protein 1 (SERTAD1) Interacts with Classical Swine Fever Virus Structural Glycoprotein E2, Which Is Involved in Virus Virulence in Swine. Viruses.

[74] Borca M.V., Vuono E.A., Ramirez-Medina E., Azzinaro P., Berggren K.A., Singer M., Rai A., Pruitt S., Silva E.B., Velazquez-Salinas L. (2019). Structural Glycoprotein E2 of Classical Swine Fever Virus Interacts with Host Protein Dynactin Subunit 6 (DCTN6) during the Virus Infectious Cycle. J. Virol..

[75] Gladue D.P., Holinka L.G., Fernandez-Sainz I.J., Prarat M.V., O’Donnell V., Vepkhvadze N.G., Lu Z., Risatti G.R., Borca M.V. (2011). Interaction between Core protein of classical swine fever virus with cellular IQGAP1 protein appears essential for virulence in swine. Virology.

[76] Sheng C., Yao Y., Chen B., Wang Y., Chen J., Xiao M. (2013). RNA helicase is involved in the expression and replication of classical swine fever virus and interacts with untranslated region. Virus Res..

[77] Liu X., Wang X., Wang Q., Luo M., Guo H., Gong W., Tu C., Sun J. (2018). The eukaryotic translation initiation factor 3 subunit E binds to classical swine fever virus NS5A and facilitates viral replication. Virology.

[78] Gladue D.P., O’Donnell V., Fernandez-Sainz I.J., Fletcher P., Baker-Branstetter R., Holinka L.G., Sanford B., Carlson J., Lu Z., Borca M.V. (2014). Interaction of structural core protein of classical swine fever virus with endoplasmic reticulum-associated degradation pathway protein OS9. Virology.

[79] Chengcheng Z., Fuxi Z., Mengjiao G., Baoyang R., Xuefeng W., Yantao W., Xiaorong Z. (2020). CSFV protein NS5A activates the unfolded protein response to promote viral replication. Virology.

[80] Lv H., Dong W., Guo K., Jin M., Li X., Li C., Zhang Y. (2018). Tumor Necrosis Factor Receptor-Associated Factor 5 Interacts with the NS3 Protein and Promotes Classical Swine Fever Virus Replication. Viruses.

[81] Wang J., Chen S., Liao Y., Zhang E., Feng S., Yu S., Li L.F., He W.R., Li Y., Luo Y. (2017). Correction for Wang et al., “Mitogen-Activated Protein Kinase Kinase 2, a Novel E2-Interacting Protein, Promotes the Growth of Classical Swine Fever Virus via Attenuation of the JAK-STAT Signaling Pathway”. J. Virol..

[82] Wang J., Chen S., Liao Y., Zhang E., Feng S., Yu S., Li L.F., He W.R., Li Y., Luo Y. (2016). Mitogen-Activated Protein Kinase Kinase 2, a Novel E2-Interacting Protein, Promotes the Growth of Classical Swine Fever Virus via Attenuation of the JAK-STAT Signaling Pathway. J. Virol..

[83] Pei J., Zhao M., Ye Z., Gou H., Wang J., Yi L., Dong X., Liu W., Luo Y., Liao M. (2014). Autophagy enhances the replication of classical swine fever virus in vitro. Autophagy.

[84] Fan S., Wu K., Luo C., Li X., Zhao M., Song D., Ma S., Zhu E., Chen Y., Ding H. (2019). Dual NDP52 Function in Persistent CSFV Infection. Front. Microbiol..

[85] Qian G., Lv H., Lin J., Li X., Lv Q., Wang T., Zhang J., Dong W., Guo K., Zhang Y. (2018). FHC, an NS4B-interacting Protein, Enhances Classical Swine Fever Virus Propagation and Acts Positively in Viral Anti-apoptosis. Sci. Rep..

[86] Li H., Zhang C., Cui H., Guo K., Wang F., Zhao T., Liang W., Lv Q., Zhang Y. (2016). FKBP8 interact with classical swine fever virus NS5A protein and promote virus RNA replication. Virus Genes.

[87] Guo K., Li H., Tan X., Wu M., Lv Q., Liu W., Zhang Y. (2017). Molecular chaperone Jiv promotes the RNA replication of classical swine fever virus. Virus Genes.

[88] Zhang C., Kang K., Ning P., Peng Y., Lin Z., Cui H., Cao Z., Wang J., Zhang Y. (2015). Heat shock protein 70 is associated with CSFV NS5A protein and enhances viral RNA replication. Virology.

[89] Shi Z., Sun J., Guo H., Yang Z., Ma Z., Tu C. (2013). Down-regulation of cellular protein heme oxygenase 1 inhibits proliferation of classical swine fever virus in PK-15 cells. Virus Res..

[90] Gong X., Li X., You X., Hu A., Liu M., Yao H., He J., Zhang X., Ning P. (2020). AIF1 was identified as an up-regulated gene contributing to CSFV Shimen infection in porcine alveolar macrophage 3D4/21 cells. PeerJ.

[91] Gladue D.P., Baker-Bransetter R., Holinka L.G., Fernandez-Sainz I.J., O’Donnell V., Fletcher P., Lu Z., Borca M.V. (2014). Interaction of CSFV E2 protein with swine host factors as detected by yeast two-hybrid system. PLoS ONE.

[92] Kang K., Guo K., Tang Q., Zhang Y., Wu J., Li W., Lin Z. (2012). Interactive cellular proteins related to classical swine fever virus non-structure protein 2 by yeast two-hybrid analysis. Mol. Biol. Rep..

[93] Zhang C., He L., Kang K., Chen H., Xu L., Zhang Y. (2014). Screening of cellular proteins that interact with the classical swine fever virus non-structural protein 5A by yeast two-hybrid analysis. J. Biosci..

[94] Shi B.J., Liu C.C., Zhou J., Wang S.Q., Gao Z.C., Zhang X.M., Zhou B., Chen P.Y. (2016). Entry of Classical Swine Fever Virus into PK-15 Cells via a pH-, Dynamin-, and Cholesterol-Dependent, Clathrin-Mediated Endocytic Pathway That Requires Rab5 and Rab7. J. Virol..

[95] Li S., Wang J., Yang Q., Naveed Anwar M., Yu S., Qiu H.J. (2017). Complex Virus-Host Interactions Involved in the Regulation of Classical Swine Fever Virus Replication: A Minireview. Viruses.

[96] Vrancken R., Paeshuyse J., Haegeman A., Puerstinger G., Froeyen M., Herdewijn P., Kerkhofs P., Neyts J., Koenen F. (2008). Imidazo[4,5-c]pyridines inhibit the in vitro replication of the classical swine fever virus and target the viral polymerase. Antivir. Res..

[97] Vrancken R., Haegeman A., Paeshuyse J., Puerstinger G., Rozenski J., Wright M., Tignon M., Le Potier M.F., Neyts J., Koenen F. (2009). Proof of concept for the reduction of classical swine fever infection in pigs by a novel viral polymerase inhibitor. J. Gen. Virol..

[98] Vrancken R., Haegeman A., Dewulf J., Paeshuyse J., Puerstinger G., Tignon M., Le Potier M.F., Neyts J., Koenen F. (2009). The reduction of CSFV transmission to untreated pigs by the pestivirus inhibitor BPIP: A proof of concept. Vet. Microbiol..

[99] Baginski S.G., Pevear D.C., Seipel M., Sun S.C., Benetatos C.A., Chunduru S.K., Rice C.M., Collett M.S. (2000). Mechanism of action of a pestivirus antiviral compound. Proc. Natl. Acad. Sci. USA.

[100] Musiu S., Pürstinger G., Stallinger S., Vrancken R., Haegeman A., Koenen F., Leyssen P., Froeyen M., Neyts J., Paeshuyse J. (2014). Substituted 2,6-bis(benzimidazol-2-yl)pyridines: A novel chemical class of pestivirus inhibitors that targets a hot spot for inhibition of pestivirus replication in the RNA-dependent RNA polymerase. Antivir. Res..

[101] Jun H.R., Pham C.D., Lim S.I., Lee S.C., Kim Y.S., Park S., Kwon M.H. (2010). An RNA-hydrolyzing recombinant antibody exhibits an antiviral activity against classical swine fever virus. Biochem. Biophys. Res. Commun..

[102] Zhou B., Liu K., Wei J.C., Mao X., Chen P.Y. (2010). Inhibition of replication of classical swine fever virus in a stable cell line by the viral capsid and Staphylococcus aureus nuclease fusion protein. J. Virol. Methods.

[103] Krol E., Pastuch-Gawolek G., Nidzworski D., Rychlowski M., Szeja W., Grynkiewicz G., Szewczyk B. (2014). Synthesis and antiviral activity of a novel glycosyl sulfoxide against classical swine fever virus. Bioorg. Med. Chem..

[104] Krol E., Wandzik I., Szeja W., Grynkiewicz G., Szewczyk B. (2010). In vitro antiviral activity of some uridine derivatives of 2-deoxy sugars against classical swine fever virus. Antivir. Res..

[105] Tyborowska J., Zdunek E., Szewczyk B. (2007). Effect of N-glycosylation inhibition on the synthesis and processing of classical swine fever virus glycoproteins. Acta Biochim. Pol..

[106] Pastuch-Gawolek G., Chaubey B., Szewczyk B., Krol E. (2017). Novel thioglycosyl analogs of glycosyltransferase substrates as antiviral compounds against classical swine fever virus and hepatitis C virus. Eur. J. Med. Chem..

[107] Krol E., Pastuch-Gawolek G., Chaubey B., Brzuska G., Erfurt K., Szewczyk B. (2018). Novel Uridine Glycoconjugates, Derivatives of 4-Aminophenyl 1-Thioglycosides, as Potential Antiviral Compounds. Molecules.

[108] Chun X., Wu D., Wu W., Wan J., Wang L., Yang T., Wang Q., Ning Y. (2005). Cloning and expression of interferon-alpha/gamma from a domestic porcine breed and its effect on classical swine fever virus. Vet. Immunol. Immunopathol..

[109] Fernandez-Sainz I., Ramanathan P., O’Donnell V., Diaz-San Segundo F., Velazquez-Salinas L., Sturza D.F., Zhu J., de los Santos T., Borca M.V. (2015). Treatment with interferon-alpha delays disease in swine infected with a highly virulent CSFV strain. Virology.

[110] Zhang X., Jing J., Li W., Liu K., Shi B., Xu Q., Ma Z., Zhou B., Chen P. (2015). Porcine Mx1 fused to HIV Tat protein transduction domain (PTD) inhibits classical swine fever virus infection in vitro and in vivo. BMC Vet. Res..

[111] Yu S., Yin C., Song K., Li S., Zheng G.L., Li L.F., Wang J., Li Y., Luo Y., Sun Y. (2019). Engagement of cellular cholesterol in the life cycle of classical swine fever virus: Its potential as an antiviral target. J. Gen. Virol..

[112] Liang X.D., Zhang Y.N., Liu C.C., Chen J., Chen X.N., Sattar Baloch A., Zhou B. (2019). U18666A inhibits classical swine fever virus replication through interference with intracellular cholesterol trafficking. Vet. Microbiol..

[113] Pei J., Deng J., Ye Z., Wang J., Gou H., Liu W., Zhao M., Liao M., Yi L., Chen J. (2016). Absence of autophagy promotes apoptosis by modulating the ROS-dependent RLR signaling pathway in classical swine fever virus-infected cells. Autophagy.

[114] Ma S., Mao Q., Chen W., Zhao M., Wu K., Song D., Li X., Zhu E., Fan S., Yi L. (2019). Serum Lipidomics Analysis of Classical Swine Fever Virus Infection in Piglets and Emerging Role of Free Fatty Acids in Virus Replication In Vitro. Front. Cell. Infect. Microbiol..

[115] Zhu E., Chen W., Qin Y., Ma S., Fan S., Wu K., Li W., Fan J., Yi L., Ding H. (2019). Classical Swine Fever Virus Infection Induces Endoplasmic Reticulum Stress-Mediated Autophagy to Sustain Viral Replication in vivo and in vitro. Front. Microbiol..

[116] He W., Xu H., Gou H., Yuan J., Liao J., Chen Y., Fan S., Xie B., Deng S., Zhang Y. (2017). CSFV Infection Up-Regulates the Unfolded Protein Response to Promote Its Replication. Front. Microbiol..

[117] Knoetig S.M., McCullough K.C., Summerfield A. (2002). Lipopolysaccharide-induced impairment of classical swine fever virus infection in monocytic cells is sensitive to 2-aminopurine. Antivir. Res..

[118] Cao Z., Zheng M., Lv H., Guo K., Zhang Y. (2018). Tissue expression of Toll-like receptors 2, 3, 4 and 7 in swine in response to the Shimen strain of classical swine fever virus. Mol. Med. Rep..

[119] Chen Y., Fan S., Zhao M., Wu K., Zhu E., Ma S., He W., Deng S., Xu H., Zhang J. (2020). MG132 Attenuates the Replication of Classical Swine Fever Virus in vitro. Front. Microbiol..

[120] Freitas T.R., Caldas L.A., Rebello M.A. (1998). Prostaglandin A1 inhibits replication of classical swine fever virus. Mem. Inst. Oswaldo Cruz.

[121] Freitas T.R., Caldas L.A., Rebello M.A. (2003). Effect of prostaglandin A1 in porcine cells persistently infected with classical swine fever virus. J. Basic Microbiol..

[122] Yin L., Luo Y., Liang B., Wang F., Du M., Petrenko V.A., Qiu H.J., Liu A. (2014). Specific ligands for classical swine fever virus screened from landscape phage display library. Antivir. Res..

[123] Vuono E.A., Ramirez-Medina E., Holinka L.G., Baker-Branstetter R., Borca M.V., Gladue D.P. (2019). Interaction of Structural Glycoprotein E2 of Classical Swine Fever Virus with Protein Phosphatase 1 Catalytic Subunit Beta (PPP1CB). Viruses.

[124] Fire A., Xu S., Montgomery M.K., Kostas S.A., Driver S.E., Mello C.C. (1998). Potent and specific genetic interference by double-stranded RNA in Caenorhabditis elegans. Nature.

[125] Xu X., Guo H., Xiao C., Zha Y., Shi Z., Xia X., Tu C. (2008). In vitro inhibition of classical swine fever virus replication by siRNAs targeting Npro and NS5B genes. Antivir. Res..

[126] Porntrakulpipat S., Supankong S., Chatchawanchonteera A., Pakdee P. (2010). RNA interference targeting nucleocapsid protein (C) inhibits classical swine fever virus replication in SK-6 cells. Vet. Microbiol..

[127] Li J., Dai Y., Liu S., Guo H., Wang T., Ouyang H., Tu C. (2011). In vitro inhibition of CSFV replication by multiple siRNA expression. Antivir. Res..

[128] Wu C.W., Chien M.S., Huang C. (2013). Characterization of the swine U6 promoter for short hairpin RNA expression and its application to inhibition of virus replication. J. Biotechnol..

[129] Li J., Guo H., Shi Z., Tu C. (2010). In vitro inhibition of CSFV replication by retroviral vector-mediated RNA interference. J. Virol. Methods.

[130] Shen L., Li Y., Chen J., Li C., Huang J., Luo Y., Sun Y., Li S., Qiu H.J. (2014). Generation of a recombinant classical swine fever virus stably expressing the firefly luciferase gene for quantitative antiviral assay. Antivir. Res..

[131] Xie Z., Pang D., Yuan H., Jiao H., Lu C., Wang K., Yang Q., Li M., Chen X., Yu T. (2018). Genetically modified pigs are protected from classical swine fever virus. PLoS Pathog..

[132] Lu C., Pang D., Li M., Yuan H., Yu T., Huang P., Li J., Chen X., Jiao H., Xie Z. (2019). CRISPR/Cas9-Mediated Hitchhike Expression of Functional shRNAs at the Porcine miR-17-92 Cluster. Cells.

[133] Simmonds P., Becher P., Bukh J., Gould E.A., Meyers G., Monath T., Muerhoff S., Pletnev A., Rico-Hesse R., Smith D.B. (2017). ICTV Virus Taxonomy Profile: Flaviviridae. J. Gen. Virol..

[134] Dai Z., Wu R., Zhao Y.C., Wang K.K., Huang Y.Y., Yang X., Xie Z.C., Tu C.C., Ouyang H.S., Wang T.D. (2014). Early lethality of shRNA-transgenic pigs due to saturation of microRNA pathways. J. Zhejiang Univ. Sci. B.

[135] Wen W., He Z., Jing Q., Hu Y., Lin C., Zhou R., Wang X., Su Y., Yuan J., Chen Z. (2015). Cellular microRNA-miR-548g-3p modulates the replication of dengue virus. J. Infect..

[136] Ottosen S., Parsley T.B., Yang L., Zeh K., van Doorn L.J., van der Veer E., Raney A.K., Hodges M.R., Patick A.K. (2015). In vitro antiviral activity and preclinical and clinical resistance profile of miravirsen, a novel anti-hepatitis C virus therapeutic targeting the human factor miR-122. Antimicrob. Agents Chemother..

[137] Touret F., Gilles M., Barral K., Nougairède A., van Helden J., Decroly E., de Lamballerie X., Coutard B. (2020). In vitro screening of a FDA approved chemical library reveals potential inhibitors of SARS-CoV-2 replication. Sci. Rep..

